# Stepping up for clinical research

**DOI:** 10.1038/s41467-021-27389-z

**Published:** 2021-12-16

## Abstract

Since its inception 10 years ago Nature Communications has strived to publish papers of high quality and relevance to communities of researchers across the whole of the natural sciences. In more recent years, we have happily seen an increase in the submission and publication of clinical research studies. To serve the clinical community to the best of our ability we have clarified our *guide to authors* on what we expect to see on submission of clinical research papers and we are launching a Clinical Collection that presents under one umbrella a selection of the interesting papers we have published in this area

Transparency and rigorous reporting standards in clinical research are key to engaging all stakeholders in a fair and responsible manner and are necessary for the community at the interface between clinical and translational research to deliver on the challenges faced by medicine today. To this effect, we recently formalised our guide to authors on what we expect to see on submission of clinical research papers. These changes have been made in line with our colleagues at Nature Medicine and adhere to the standards set out by The International Committee of Medical Journal Editors - ICMJE. In particular, our guide to authors provides detailed information on our requirements for the reporting of results from interventional trials; these include: the need for prospective study registration, the submission of a study protocol and conditions for publication of interim analyses.

Prospective trial registration serves several purposes; it provides visibility to patients of trials that they could participate in, allows researchers to see what other trials are already planned/ongoing, and importantly ensures that the pre-planned health outcomes that a study aims to measure are clearly defined. The databases for clinical trial registration that have been approved by ICMJE are publicly available, searchable and available for any researcher to use.

In line with our policy, on receipt of a clinical trial paper, we verify the details of registration, including the study start and registration date; we only consider intervention trials that were registered before the study start date. We know that any clinical study protocol is a live document that is frequently amended and appreciate that these amendments do not always get added to trials registry sites; therefore we ask to see the protocol to ensure that we are considering the most up to date description of the study. Unless there is an express reason not to do so we will also share the study protocol with our reviewers and encourage its publication alongside the manuscript.

We encourage submission of studies from across the whole of clinical medicine including, but not limited to, cancer, cardiovascular disease, diabetes, diet, immunology, infectious diseases, metabolism, neuroscience as well as interventions addressing public and global health issues.

Both the trial registration sites and study protocols should provide detailed lists of the intended primary and secondary outcomes of clinical studies. We expect that all of the primary outcomes of a study would be reported, unless they have already been reported elsewhere and the submitted study is exploring additional facets of the datasets. With regards to secondary outcomes, while we do not necessarily expect that these will be described, we consider it good practice to include results of all secondary outcomes when one secondary outcome is presented. We discourage the partial reporting of secondary outcomes or adhoc outcomes as this may result in the selective reporting of results. Additional results based on the data collected within the trial framework are also permitted if these are clearly indicated as ad hoc analyses.

In line with ICMJE recommendations, trials registered from January 2019 should also include a data sharing plan from the outset. All our published manuscripts, regardless of the starting date of the trial, should describe the type of data available, when it will be available and with whom it can be shared in a Data Availability Statement.

We understand that interim reports of an intervention study may be justified, for example when a trial is still recruiting or when the full longitudinal analysis is still pending; however, to avoid selective reporting of results, we will either need to see that provision was made in the study protocol for interim reporting or documentation from, for example, a data safety monitoring board approving the interim analysis.

These policies largely apply only to interventional clinical trials; we are very happy to receive submissions from clinical trials of any phase, including randomised controlled trials. However, we’re also interested in hearing from researchers who wish to publish case reports or small case series, conditional with permission for any interim reporting. We do expect that randomised trials follow CONSORT *guidelines*, including the provision of a consort diagram, and also encourage authors of other types of clinical trials, including n of 1 trials, to follow these recommendations. More recent extensions to CONSORT have also been developed to facilitate the reporting of trials that contain the use of artificial intelligence or that report on interventions relevant to health equity.

Reporting of interventional trials with neutral results of high relevance to a particular community is also welcome. Our interest in clinical trials is not just limited to interventional studies; we also welcome observational studies, for which we encourage registration, even if retrospective. Meta-analyses and systematic reviews are also both within the scope of our journal.

We’re delighted to receive submissions of studies from across the whole of clinical medicine including, but not limited to, cancer, cardiovascular disease, diabetes, diet, immunology, infectious diseases, metabolism, neuroscience as well as interventions addressing public and global health issues.

Naturally, we continue to be interested in basic research and welcome translational research; indeed we find that many of our studies describe pre-clinical findings together with some support from clinical data. At the other end of the spectrum, we appreciate that the primary results of a trial may have been reported elsewhere, however, if addressing a problem of relevance to understanding of a disease or important to inform treatment, we are happy to consider papers detailing secondary analyses only or ad hoc analyses from data collected from a published clinical trial.

We hope that by updating our guide to authors for clinical papers we are providing clarity on what we expect from our clinical research submissions. By adhering to the principles of ICJME we are committing to publishing studies that uphold high standards for the transparent and accurate reporting of clinical data. Examples of our published clinical papers can be found with the accompanying collection. If you have any questions about the suitability of your clinical study for Nature Communications please do reach out to us with questions, our full list of editors can be found here, https://www.nature.com/ncomms/about/editors

